# Toward a multiscale modeling framework for understanding serotonergic function

**DOI:** 10.1177/0269881117699612

**Published:** 2017-04-18

**Authors:** KongFatt Wong-Lin, Da-Hui Wang, Ahmed A Moustafa, Jeremiah Y Cohen, Kae Nakamura

**Affiliations:** 1 Intelligent Systems Research Centre, School of Computing and Intelligent Systems, University of Ulster, Magee Campus, Derry~Londonderry, UK; 2 School of Systems Science, and National Key Laboratory of Cognitive Neuroscience and Learning, Beijing Normal University, Beijing, China; 3 School of Social Sciences and Psychology, and Marcs Institute for Brain and Behaviour, University of Western Sydney, Sydney, Australia; 4 Solomon H. Snyder Department of Neuroscience, Brain Science Institute, Johns Hopkins University School of Medicine, Baltimore, USA; 5 Department of Physiology, Kansai Medical University, Hirakata, Osaka, Japan

**Keywords:** Serotonin 5-HT, midbrain raphe nucleus, neural circuit, multiscale computational model

## Abstract

Despite its importance in regulating emotion and mental wellbeing, the complex structure and function of the serotonergic system present formidable challenges toward understanding its mechanisms. In this paper, we review studies investigating the interactions between serotonergic and related brain systems and their behavior at multiple scales, with a focus on biologically-based computational modeling. We first discuss serotonergic intracellular signaling and neuronal excitability, followed by neuronal circuit and systems levels. At each level of organization, we will discuss the experimental work accompanied by related computational modeling work. We then suggest that a multiscale modeling approach that integrates the various levels of neurobiological organization could potentially transform the way we understand the complex functions associated with serotonin.

## Introduction

Serotonin (5-hydroxytryptamine; 5-HT) neurons are clustered into rostral and caudal groups. The rostral group is located at the mesencephalon and rostral pons, including the midbrain raphe nuclei with major projections to the forebrain and are critical for the regulation of various functions, including emotion and decision-making (Müller and Jacobs, 2009). The caudal group is located from the caudal pons to the caudal portion of the medulla oblongata, with major projections to the brainstem and the spinal cord (Ding et al., 2003; Hornung, 2003). The effects of 5-HT on brain functions is mediated through its release and reuptake, and its 14 receptor subtypes throughout the brain (Barnes and Sharp, 1999). Genetic variation in 5-HT can affect emotional processing in animals and humans, and research has reported links between 5-HT and vulnerability to anxiety and depression (Müller and Jacobs, 2009). Although 5-HT-targeted drugs can have important therapeutic effects on various disorders (e.g. depression and post-traumatic stress disorders), such agents currently lack the efficacy and tolerability required, and further improvement is needed (Nichols and Nichols, 2008). This is largely due to the still unknown mechanisms that 5-HT exerts on brain systems.

Computational modeling and mathematical theories are useful tools to provide quantitative and conceptual understanding of observed experimental phenomena, while offering predictions for future tests (Abbott, 2008). Despite decades of research on the 5-HT system, its computational roles are still not completely known (Nakamura and Wong-Lin, 2014). Theories on the role of 5-HT in health and disease have been separately developed at different levels of biological descriptions, with efforts largely focused on either models with high biological details such as intracellular signaling mechanisms or those on cognitive and behavioral aspects such as decision-making (e.g. Doya, 2002; Zhou et al., 2014). With technological advancements, levels intermediate to these are more readily studied, revealing highly heterogeneous, complex and multifunctional aspects of the 5-HT system (e.g. Cohen et al., 2015; Muzerelle et al., 2016; Okaty et al., 2015). These experimental findings present challenges in modeling and understanding 5-HT functions.

In this paper, we will review existing experimental and computational modeling work on the 5-HT system at various levels of description, from intracellular through neuronal circuit to systems and behavioral levels (sections 2–5). Toward the end (section 6), we suggest, with some examples, that a multiscale computational modeling framework that integrates across multiple scales of 5-HT functions could potentially embrace the new types of complex data and further illuminate 5-HT functions. The paper will have a computational modeling focus, and biologically-based models will be emphasized as they have previously received less attention. We will not be able to cover the wide spectrum of studies on 5-HT system. For further information regarding detailed experimental work, we shall refer the readers to other comprehensive reviews on the 5-HT system (e.g. Jacobs and Azmitia, 1992; Barnes and Sharp, 1999; Celada et al., 2013; Müller and Jacobs, 2009; Smythies, 2005). For more extensive discussions on modeling neuromodulation and their effects on cognition, we refer the readers to insightful reviews, such as those by Fellous and Linster (1998), Doya (2002), Dayan (2012) and Marder (2012).

## Intracellular signaling processes

### 5-HT presynaptic terminals

**Figure 1.**
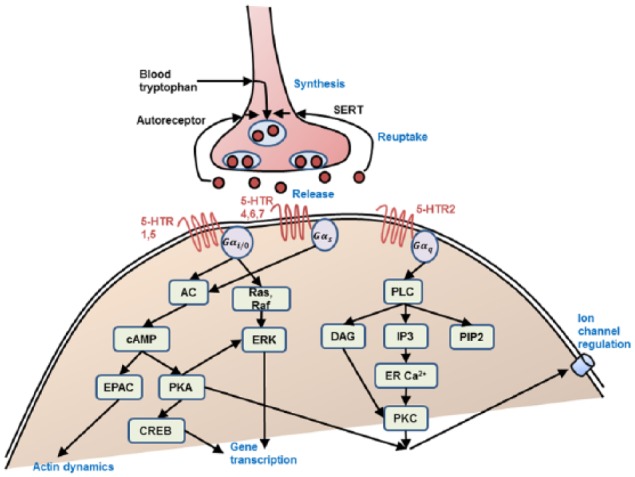
Schematic of the 5-HT presynaptic terminal processes and signal transduction pathways. Abbreviations as defined in main text. Only metabotropic 5-HT receptors are shown. Compared with Table 1. 5-HT: 5-hydroxytryptamine; SERT: serotonin reuptake transporter; Gα_i_/Gα_o_, Gα_s_, Gα_q_: isoforms of the α subunits of G protein-coupled receptors; AC: adenylyl cyclases; cAMP: cyclic adenosine monophosphate; EPAC: exchange proteins activated by cAMP; PKA: protein kinase A; CREB: cAMP response element-binding protein; Raf: rapidly accelerated fibrosarcoma kinase; ERK: extracellular signal regulated kinase; PLC: phospholipase C; IP3: inositol 1,4,5-trisphosphate; DAG: diacylglycerol; PIP2: phospholipid phosphatidylinositol 4,5-bisphosphate; ER: endoplasmic reticulum; PKC: protein kinase C.

In the presynaptic terminals, 5-HT is synthesized and stored via a series of biochemical reactions beginning with the uptake of tryptophan. With sufficient presynaptic 5-HT terminal excitability and spiking activity, 5-HT can be released into the extracellular space. The released 5-HT may bind to postsynaptic 5-HT receptors (see below), be metabolized to 5-hydroxyindoleacetic acid (5-HIAA) via monoamine oxidase (MAO), removed through diffusion, or reabsorbed back to the presynaptic terminal via serotonin reuptake transporter (SERT) (Figure 1). Polymorphisms and alterations in the *SERT* gene have been linked to depression and mood disorders (Ansorge et al., 2004; Caspi, 2003; Heils et al., 1996; White et al., 2005). In addition, 5-HT1A autoreceptors can regulate presynaptic neuronal firing rates, while 5-HT1B autoreceptors decrease synthesis and release with increasing extracellular 5-HT concentration, with over-expression of 5-HT1A autoreceptors implicated in reducing serotonergic neurotransmission, and associated with major depression and suicide (Albert et al., 2011). In fact, 5-HT neurons’ unique identity arises from the co-expressing of genes including those directing 5-HT synthesis, reuptake, vesicular transport, autoreceptor signaling and metabolism, and alterations in the transcription regulatory networks governing these processes can lead to physiological and behavioral pathogenesis (Deneris and Wyler, 2012).

**Table 1.** Some examples of serotonin (5-HT) modulatory effects on neurons and synapses due to various 5-HT receptor subtypes. Abbreviations as defined in main text. ↑ and ↓ denote an increase and decrease, respectively. Compared with Figure 1.

Receptor subtype	Associated G-protein	Second messengers	Modulatory effects
**5-HT1**	Gα_i_/Gα_o_	cAMP,PKA	↓cAMP, open K^+^ channels, ↓AMPA, ↓NMDA, ↓GABA,
**5-HT2**	Gα_q_	PLC,IP3, DAG, PKC	↑cAMP, open Ca^2+^channel, ↑AMPA, ↑NMDA, ↓GABA
**5-HT3**	Ion channels	Ligand-gated Na^+^ and K^+^ channels	Fast excitatory postsynaptic potential
**5-HT4**	Gα_s_	cAMP,PKA	↑cAMP
**5-HT5**	Gα_i_/Gα_o_		↓cAMP
**5-HT6**	Gα_s_	cAMP,PKA	↑cAMP
**5-HT7**	Gα_s_	cAMP,PKA	↑cAMP

5-HT: 5-hydroxytryptamine; Gα_i_/Gα_o_, Gα_s_, Gα_q_: isoforms of the α subunits of G protein-coupled receptors; cAMP: cyclic adenosine monophosphate; PKA: protein kinase A; PKC: protein kinase C; DAG: diacylglycerol; PLC: phospholipase C; IP3: inositol 1,4,5-trisphosphate; AMPA: α-amino-3-hydroxy-5-methyl-4-isoxazolepropionic acid; NMDA: N-Methyl-D-aspartic acid; GABA: gamma-Aminobutyric acid.

Pharmacological drugs can directly affect these presynaptic signaling processes. For example, selective serotonin reuptake inhibitors (SSRIs) and tricyclic antidepressants (TCAs) can block SERT on cell bodies, including presynaptic terminals, thus raising extracellular 5-HT concentration levels (Casanovas and Artigas, 1996; Gartside et al., 1995; Gillman, 2007; Tatsumi et al., 1997). Chronic treatments can lead to the desensitization of 5-HT1A autoreceptors (affecting 5-HT neuronal excitability) and downregulation of *SERT* mRNA (Adell et al., 2002; Benmansour et al., 2002; Mizra et al., 2007). Variations in *SERT* gene expression may be responsible for variability in antidepressant response (Porcelli et al., 2011). MAO inhibitors play a similar role with regard to increasing extracellular 5-HT by reducing the catabolism of 5-HT. However, TCAs and MAO inhibitors also affect other monoamines such as dopamine and norepinephrine and other receptor types, and thus their effects are more complex (Gillman, 2007; Shulman et al., 2013).

### Models of 5-HT presynaptic terminals

Given the important direct relationships between intracellular signaling and drugs, it is imperative to understand their mechanisms more deeply, using in silico models. Currently, there are few computational models of 5-HT presynaptic signaling. Stoltenberg and Nag (2010) made use of control theory with differential and difference equations in developing a dynamical systems model. This model can simulate 5-HT neuronal firing rate due to tryptophan hydroxylase 2 (TPH2) and serotonin-transported-linked polymorphic region (5-HTTLPR) genotypes, and cerebrospinal fluid levels of the 5-HT metabolite 5-hydroxyin-doleacetic acid (CSF 5-HIAA). A biologically realistic mathematical model was developed by Best et al. (2010), based on previous work modeling the dopaminergic presynaptic terminal (Best et al., 2009). This model is constrained by experimental data, and consists of nine coupled nonlinear differential equations, describing the kinetic dynamics of the interacting substrates. Specific functions of the velocities could be derived via Michaelis–Menten kinetics. The model’s aim was to explore various hypotheses on 5-HT homeostasis and signaling. They include effects due to tryptophan (food intake), autoreceptor effects, acute dose of SSRIs, and polymorphisms of gene expressions.

Based on this model by Best et al. (2009), Flower and Wong-Lin (2014) used perturbation techniques to tease apart the relative dynamical timescales and relationship strengths among the substrates. This led to determining key relationships among the interacting substrates in the original full model, and allowed the reduction of the original model into simpler fast and slow versions. For example, the approximated reduced fast model could be described only by the relatively faster dynamics of the vesicular and extracellular 5-HT concentration levels, treating the rest of the substrates’ dynamics to be relatively constant. The fast reduced model, with only two differential equations to describe substrates’ dynamics, was able to substantially speed up the computational processing speed. This improvement becomes even more substantial when simulated with ~100,000 neurons, about the total number of 5-HT-containing neurons in the human brain (Flower and Wong-Lin, 2014).

While extracellular 5-HT concentration levels can be measured by traditional microdialysis methods, the temporal dynamics of the release and reuptake of extracellular 5-HT can be experimentally captured using voltammetry techniques (Bunin et al., 1998; Dankowski, and Wightman, 2013; Hashemi et al., 2011). Models based on such data have recently been developed, assuming the uptake kinetics of the extracellular 5-HT to follow the Michaelis–Menten equation, and fitted to the voltammetry data in tissue slice preparation (Bunin et al., 1998; Daws et al., 2005). The model has since been adopted into neural models in which neural firing can directly stimulate the increase in extracellular 5-HT level (Best et al., 2011; Flower and Wong-Lin, 2014; Joshi, 2014; Joshi et al., 2011, 2015, 2017). Using in vivo fast scan cyclic voltammetry, Wood et al. (2014) developed a more complex computational model to capture two distinct 5-HT reuptake mechanisms (one with high affinity and low efficiency and another with low affinity and high efficiency), and a rapid inhibitory autoreceptor control mechanism.

Computational models at the 5-HT presynaptic terminals at different levels of complexity have been developed. The generality of these models allow them to be applied to various brain areas innervated by 5-HT terminals. Importantly, there exist simpler models that are scalable (e.g. to the neuronal circuit level).

### 5-HT receptor signal transduction pathways

Rather early on, 5-HT was known to be an integral neuromodulator for learning and memory. In particular, due to its small nervous system, the marine mollusk *Aplysia* has been used as a powerful model system for nonassociative learning, such as habituation, dishabituation, and sensitization (Carew et al., 1971; Pinsker et al., 1970, 1973), and associative learning, such as classical, operant, and fear conditioning (Brembs et al., 2002; Carew et al., 1981, 1983; Lechner et al., 2000a, 2000b; Walters et al., 1981). Sensitization requires 5-HT-dependent synaptic plasticity from sensory to motor neurons (Brunelli et al., 1976; Castellucci and Kandel, 1976; MacKey et al., 1989; Marinesco and Carew, 2002). Specifically, the memory for sensitization exhibits distinct temporal phases: short-term, intermediate-term, and long-term sensitization (STS, ITS, LTS). ITS requires protein synthesis but not ribonucleic acid (RNA) synthesis, while LTS requires both protein and RNA synthesis.

These effects require knowledge of how the released 5-HT exerts its effects through a variety of membrane-bounded receptors, namely, ligand-gated ion channels and metabotropic 5-HT receptors (Maejima et al., 2013; Roth, 2006). The effects are complex and multifaceted, and hence deserve further attention. The 5-HT ligand-gated ion channels 5-HT3A and 5-HT3B are nonspecific cation channels and elicit fast excitatory postsynaptic potentials, while the metabotropic 5-HT receptors, with 7 transmembrane domains, are grouped into 6 families and 14 distinct subtypes with the heterotrimeric G-protein-coupled receptors (GPCRs) (Bockaert et al., 2006).

The heterotrimeric G-proteins are composed of the α and the dimeric βγ subunits. When the α subunit is bound to guanosine diphosphate (GDP), α and βγ subunits will form the inactive heterotrimeric complex (Magali, 2012; McCudden et al., 2005; Siehler and Milligan, 2011). In the presence of 5-HT and the GPCRs activated, GDP will be replaced by guanosine triphosphate (GTP) and leads to the dissociation of G-proteins, not only activating the α subunit but also liberating the βγ subunit (Millan et al., 2008). The active α subunit and βγ subunit can relay information to different downstream signaling pathways (McCudden et al., 2005). There are several isoforms of the α subunit associated with 5-HT: Gα_i_/Gα_o_, Gα_s_, and Gα_q_. For example, activated Gα_i_ subunits inhibit the adenylyl cyclases (AC), the active Gα_s_ subunits stimulate AC, activated Gα_q_ subunits stimulate phospholipase Cβ (PLCβ), and activated Gα_o_ subunits often lead to opening of K^+^ channels and closing of Ca^2+^ channels (Figure 1). The liberated βγ dimeric subunits not only can regulate ion channels such as G-protein gated inward rectifier channels (GIRK or Kir 3 channels) as well as Ca^2+^ channels, but also regulate kinase and small G-proteins such as βγ **-**mediated stimulation of extracellular signal regulated kinase (ERK) and mitogen activated protein kinases (MAPKs), Ras proteins and PLCβ (Berridge, 2014; McCudden et al., 2005).

AC, regulated by Gα_s_/Gα_i_, can convert ATP into the second messenger cyclic adenosine monophosphate (cAMP) using the catalytic regions on its larger cytoplasmic domains C1 and C2. The cAMP acts through three effector systems: protein kinase A (PKA), exchange proteins activated by cAMP (EPACs), and components of other signaling pathways. After cAMP binds to the regulatory subunits of PKA, the catalytic subunits of PKA can phosphorylate downstream target proteins to regulate specific cellular processes (Figure 1). The effects include: (i) enhancing gene transcription associated with formation of long-term memory regulated by cAMP response element-binding protein (CREB); (ii) increasing NMDA and AMPA receptor-mediated synaptic currents, and decreasing gamma-aminobutyric acid (GABA) receptor-mediated synaptic currents by phosphorylation of their subunits to control the receptors’ properties and their synaptic trafficking underlying plasticity; and (iii) modulating voltage-gated sodium, potassium, and calcium ion channels. EPACs bind to cAMP with high affinity and activate the Ras super family small GTPases Rap1 and Rap2, which can activate PLC, open the cyclic nucleotide-gated channels, and control the actin dynamics of cells (Cheng et al., 2008). The third kinds of effectors of cAMP are cyclic GMP phosphodiesterase (PDE) and Ca^2+^ channels Cav1.1 and Cav1.2. SSRIs can act on signal pathways such as cAMP on the postsynaptic neuronal cell, which can in turn release brain-derived neurotrophic factor that can enhance the growth and survival of neurons and synapses (Schwindinger and Robishaw, 2001).

PLCβ, stimulated by αq and βγ subunits, can cleave the phospholipid phosphatidylinositol 4,5-bisphosphate (PIP2) into two kinds of second messenger: soluble inositol 1,4,5-trisphosphate (IP3) and membrane-adhering diacylglycerol (DAG). IP3 diffuses into the cytosol and releases Ca^2+^ from the endoplasmic reticulum. The DAG activates the enzyme protein kinase C (PKC), where the classic PKCs (PKCα, PKCβ and PKCγ) require both Ca^2+^ and DAG, whereas novel PKCs (PKCδ, PKCϵ and PKCη) are Ca^2+^-independent (Berridge, 2014; Turner et al., 2006).

To summarize, metabolic 5-HT receptors coupled to different isoforms of G-proteins can evoke different intracellular signaling pathways, modulating neuronal and synaptic activities. Table 1 provides some examples of the various complex modulatory effects on cells and synapses due to 5-HT receptor subtypes.

### Models of signaling pathways and affected currents

The Michaelis–Menten kinetics formalism and the law of mass action approach are often used in modeling the biochemical signaling pathways (Keener and Sneyd, 2008; Michaelis and Menten, 1913). For example, to understand the interplay between 5-HT receptor subtypes on intracellular signaling pathway dynamics, a comprehensive mathematical model of 5-HT1A and 5-HT2A receptor-activated ERK pathways was developed by Chang et al. (2009). The transformation reactions were represented by the Michaelis–Menten formalism, and the dynamics of the reaction network formulated using the law of mass action. The results of this detailed mathematical model are in agreement with experimental data, showing the dominance of 5-HT2A receptor over 5-HT1A receptor in the MAPK signaling pathway, and the deleterious effects of regulator/enzymes affecting basal levels of ERK1/2. Hence, the model provides insights into the interplay of the two 5-HT receptor subtypes with potential applications in drug efficacy studies.

To more precisely formulate and test hypotheses of PKA and ERK activities in different memory learning/training protocols (short-term, intermediate-term, and long-term facilitations), Pettigrew et al. (2005) modeled 5-HT modulation on the *Aplysia* sensorimotor synapse, in which several parallel and feedback 5-HT-induced signaling pathways were simulated, including involvements of PKA, ERK and recombination-activing gene (*REG*). These led to intermediate- and long-term facilitations (ITF and LTF), which are correlated with ITS and LTS, respectively. Although this model was incomplete and did not include a gene regulatory step, it provided a qualitative representation of key biochemical processes essential for ITF and LTF of sensorimotor synapse. This model is subsequently modified with only the initial steps in the induction of LTF, plus a time delay in the phosphorylation the rapidly accelerated fibrosarcoma kinase and an inducer was added (Zhang et al., 2012). The modified version was used to search for a training protocol to maximize PKA–ERK interactions to enhance LTF. Later, to understand a previously unappreciated role for a PKC-dependent processes in the maintenance of STF, Zhou et al. (2014) modeled PKC signaling, in parallel with PKA mechanisms, to demonstrate that PKC was sufficient for STF at non-depressed synapses and the facilitation of depressed synapses.

In contrast with modeling the 5-HT metabotropic receptors, modeling the ligand-based 5-HT3 receptor largely comes from quantitative molecular analysis and homology modeling of the 5-HT3 receptor binding affinity, structure and dynamics (Menziani et al., 2001; Reeves et al., 2003; Schmidt and Peroutka, 1989). These modeling techniques are more complex, which required not only differential equations, but also other theories and methods in physics and chemistry (e.g. quantum mechanics).

The above examples show that computational models of 5-HT intracellular signaling via its various receptor subtypes can provide quantitative insights into the various effects caused by 5-HT receptors and the underlying biochemical pathways. Importantly, some of these pathways can be perturbed by drugs, and therefore, these models are highly useful for computer-aided drug discovery and development. However, the models have several parameters and complex pathway dynamics, and therefore remain a challenge in terms of scalability (e.g. bridging to the neuronal circuit level and beyond). In the next section, we will discuss how by directly modeling the effects (e.g. ion channel or current modulation), some of these complexities can be circumvented, providing a plausible solution.

## Neuronal properties

### Diversity of neuronal properties

5-HT neurons are largely found in the raphe nuclei of the brain (Müller and Jacobs, 2009). A major challenge of conventional single-unit recording is the identification of the chemical characteristics associated with the recorded neurons. For example, a substantial proportion of dorsal raphe nucleus (DRN) neurons are serotonergic: 30% in rats (Descarries et al., 1975), 70% of medium-sized DRN neurons in cats (Wiklund et al., 1981), and 70% in humans (Baker et al., 1991); however, the DRN also includes neurons containing other neurotransmitters, such as GABA, glutamate, dopamine, noradrenaline/norepinephrine and peptides (e.g. cholecystokinin, somatostatin, enkephalin, galanin, substance P, and neurotensin) (Fu et al., 2010; Jacobs and Azmitia, 1992; Kohler and Steinbusch, 1982; Michelsen et al., 2007). GABAergic neurons form the largest population of non-5-HT neurons in the dorsal raphe (Calizo et al., 2011; Celeda et al., 2001; Varga et al., 2001, 2003). However, there is little coexistence of GABA and 5-HT in the same cells (Fu et al., 2010; Shikanai et al., 2012). Dopamine, a monoamine like 5-HT, also appears to be present in DRN neurons, which are separate from 5-HT neurons (Descarries et al., 1986; Fu et al., 2010; Hökfelt et al., 1976; Yoshida et al., 1989).

The properties of neurons in the raphe nuclei are diverse and heterogeneous, including metabolism, anatomy, neurochemistry and physiology (Andrade and Haj-Dahmane, 2013; Beck et al., 2004; Fernandez et al., 2017; Gaspar and Lillesaar, 2012; Muzerelle et al., 2016; Okaty et al., 2015). Electrophysiologically, 5-HT and non-5-HT neurons are heterogeneous in terms of resting membrane potentials, input resistances, spike amplitudes and spike thresholds (Allers and Sharp, 2003; Kocsis et al., 2006; Li et al., 2001; Marinelli et al., 2004). There are also regional excitability differences among subnuclei within the DRN. For example, the lateral wings contain neurons with higher membrane excitability (Crawford et al., 2010; but see Shikanai et al., 2012). 5-HT neurons have been found to exhibit classical regular-spiking and bursting behavior (Cohen et al., 2015; Hajós et al., 1996, 2007; Hajós and Sharp, 1996; Kirby et al., 2003; Li et al., 2001) and are also associated with a characteristic slow after-hyperpolarization (AHP) (Kirby et al., 2003). The variety of 5-HT neuronal spiking behavior has been suggested to be due to the interplay among multiple ion channel currents (Aghajanian and Sanders-Bush, 2002).

### Neuronal models

Despite the wealth of electrophysiological data accumulated over the decades, the membrane excitability and spike generation of 5-HT neurons have only recently been modeled (Penington and Tuckwell, 2012; Tuckwell, 2013; Tuckwell and Penington, 2014). In their latest model, based on voltage-clamp data, Tuckwell and Penington (2014) used a biophysical single-compartmental 5-HT neuronal model with 11 ion channel currents and calcium dynamics. Their conductance-based model includes fast sodium current, delayed rectifier potassium current, transient potassium current, slow non-inactivating potassium current, low-threshold calcium current, two high-threshold calcium currents, small- and large-conductance potassium currents, hyperpolarization-activated cation current, and leak currents. These currents are modeled using the Hodgkin–Huxley formalism (Hodgkin and Huxley, 1952). With this model, Tuckwell and Penington were able to account for a wide variety of electrophysiological properties of 5-HT neurons. For instance, the neuronal membrane potential of the model could exhibit spontaneous periodic spiking, spike doublets, and subthreshold humps or notches as found in experiments. The model also supported the competitive functions of the transient potassium current and the low-threshold calcium current on interspike intervals. The model also can mimic the excitatory effects of adrenoreceptor ($\alpha_{1}$) effects by decreasing the potassium leak conductance and the resting membrane potential.

The neuronal spiking behavior of 5-HT neurons can also be modeled using different and simpler spiking neuronal models. In particular, Wong-Lin et al. (2011, 2012) have used the Izhikevich neuronal model (Izhikevich, 2003) that has only two coupled differential equations and four intrinsic neuronal parameters and hence is computationally efficient. Yet, it can replicate a repertoire of spiking and subthreshold membrane potential behaviors (e.g. slow periodic spiking, and spike doublets or triplets) as observed in 5-HT neurons without explicitly involving specific complex ion channel dynamics (Wong-Lin et al., 2011, 2012). This two-dimensional dynamical model is also highly conducive for rigorous mathematical (e.g. phase-plane) analysis, such that specific neuronal behavior can be easily selected without extensive brute-force model parameter searching (Izhikevich, 2007; Wong-Lin et al., 2011, 2012). A similar type of model, the adaptive exponential integrate-and-fire neuronal model (Brette and Gerstner, 2005), has also been adapted to model 5-HT neurons (Joshi, 2014; Joshi et al., 2015). Similarly, Tuckwell et al. (2015) subsequently also developed other reduced 5-HT neuronal models, namely the Fitzhugh–Nagumo (Fitzugh, 1961; Nagumo et al., 1962) and the reduced Hodgkin–Huxley model, both described by only two coupled differential equations. Despite some limitations (e.g. higher firing frequency), the general spiking behaviors are similar to those from experimental observations. Due to their lesser complexity, the models were also rigorously mathematically analyzed.

More generally, neuronal computational models can provide a platform to mimic the effects of 5-HT on the targeted areas, circumventing detailed modeling of signaling pathway processes. This can be done by directly simulating the affected conductances, currents or other neuronal properties. For example, based on voltage-clamp studies of sensory neurons in *Aplysia*, Baxter et al. (1999) used the Hodgkin–Huxley type model to describe the neuronal excitability of a sensory neuron modulated by 5-HT modulation. Specifically, the model was based on the knowledge that 5-HT induced elevation of cAMP modulated several membrane currents: (i) decreasing S current (I_K,s_) and a slow component of the Ca^2+^-activated K^+^ current (I_K,Ca-s_); (ii) decreasing the conductance and slowing the kinetics of a large, steeply voltage-dependent K^+^ current (I_K-V_); while (iii) enhancing a dihydropyridine-sensitive and slowly inactivating component of the Ca^2+^ current similar to the L-type Ca^2+^ current (I_Ca-L_). Thus, Baxter et al. (1999) mimicked PKA and PKC effects by decreasing the conductances of I_K,s_, I_K,Ca-s_, while increasing that of I_Ca-L_. They also simulated the actions of 5-HT on I_K-V_ by increasing the time constants of the activating and inactivating variables. Despite the model not incorporating explicit intracellular signaling, the model could replicate experimental data of the PKA and PKC effects on membrane currents and action potential waveform. The model also predicted the important contribution of I_K,Ca-s_ and I_Ca-L_ in the excitability of the sensory neurons, which had not been investigated prior to this work. Moreover, the model prompted further investigation of Na^+^ currents to fill the remaining discrepancy between the neuronal model behavior and experimental data.

Other models that mimicked the affected currents include Bertram (1993, 1994). Bertram (1993) modeled and mathematically analyzed how 5-HT could affect the burster R_15_ neuronal activity by modulating its subthreshold inward rectifier currents (I_R_) and negative-slope-region currents (I_NSR_) that control the neuronal bursting mechanism. The model suggested that 5-HT increases the sensitivity of the burster neuron to synaptic perturbations due to the competition between various states (stationary, bursting and the beating attractor). Bertram (1994) further reduced the model for more detailed mathematical analysis of the model.

With a similar modeling approach, Cano-Colino et al. (2014) modeled 5-HT1A and 2A effects on prefrontal cortical neurons by modulating the 5-HT induced currents. Specifically, the 5-HT1A effects on prefrontal cortical (PFC) excitatory pyramidal cells were simulated by modeling the inhibitory 5-HT modulation on some GIRK currents (I_GIRK_). In contrast, 5-HT2A exerted excitatory effects on pyramidal cells via increased in intracellular Ca^2+^, which simultaneously inhibited Ca^2+^-activated afterhyperpolarization currents (I_K,Ca_) and activated an afterdepolarization current mediated by a Ca^2+^-dependent nonselective cation channel (I_Can_). In addition, their model also included 5-HT2A modulation of prefrontal inhibitory interneurons by decreasing the conductance of their leak current. This model will be further discussed in the next section.

To allow even higher scalability in the modeling, Joshi et al. (2017) fitted a nonlinear relationship between 5-HT concentration levels and the induced currents on targeted neurons based on experimental data from electrophysiology, pharmacology and voltammetry. By shifting the nonlinear input–output functions or changing the parameter values in the Michaelis–Menten relationships, the model can simulate drug (e.g. antidepressant) effects. The modeling framework proposed was sufficiently general to model the interactions of multiple drugs and neuromodulators on multiple brain regions. Simpler and more abstract models have been developed (Jalewa et al., 2014; Joshi et al., 2011); the modulated currents in Jalewa et al. (2014) were more abstract (i.e. based on a neural firing-rate type model (Wilson and Cowan, 1972)); and Joshi et al. (2011) directly obtained the nonlinear function from 5-HT to the neuronal population firing rate without introducing any affected currents. In contrast with 5-HT modulation via its metabotropic receptors, simplified more scalable model for 5-HT3 mediated currents has only recently been modeled (Jalewa et al., 2014). These models will be further discussed in the next section.

In summary, the computational models of 5-HT neurons can be rather complex with several ion channel currents involved. However, more recent modeling work has attempted to reduce the model to allow better scalability (e.g. neuronal circuit level). Models of neurons and neural populations innervated by 5-HT can also be simulated, circumventing the need to explicitly model the complex signaling transduction pathways. This is mainly achieved through direct modeling of the affected currents in the neuronal or neuronal population models.

## Single neuronal functions and neuronal microcircuits

### Circuits and functions of 5-HT neurons

A useful way to understand 5-HT function in the brain is to measure the activation pattern of single cells (‘units’) in the raphe nuclei of animals performing behavioral tasks. This can provide rich information about the context and events encoded by DRN neurons, at least some of which secrete 5-HT, and behavioral-pharmacological experiments can then examine how 5-HT is utilized at the projection sites. Due to its high spatiotemporal resolution, single-unit recording is also beneficial for analyzing trial-by-trial changes in activity related to particular aspects of behavioral tasks.

To date, several characteristics and heterogeneity of DRN neuronal activity have emerged from single-unit recording studies in awake animals (Cohen et al., 2015; Hayashi et al., 2015; Inaba et al., 2013; Li et al., 2013, 2016; Miyazaki et al., 2011; Nakamura et al., 2008; Ranade and Mainen, 2009). First, DRN neurons respond to various task events, including visual, olfactory, and somatosensory events, to incentive events such as rewards and punishments, to movement, and to delay, which indicates that there is a wide array of inputs to the DRN. Second, different activation patterns in terms of timescale were observed; very brief responses, sensory stimuli; brief responses well-aligned to the onset of tonic activity that may last across trials, which encodes appetitive and aversive contexts (Cohen et al., 2015; Hayashi et al., 2015). Third, responses with modulations in opposite directions were observed. For example, a group of neurons exhibited an increase while others exhibited a decrease in activity during the same sensory events, such as a tail pinch (Schweimer and Ungless, 2010) or air puff (Cohen et al., 2015). Task factors such as reward value also evoked opposite direction modulations in DRN neurons (Hayashi et al., 2015; Nakamura, 2013). It is unknown how and why such mirror-imaged modulation in neuronal activity is created and utilized downstream of the information processing. The characteristics of the DRN neuronal activity described above are in strong contrast to those observed in midbrain dopamine neurons which typically exhibit phasic, not tonic, and uniformly excitatory responses (Nakamura, 2013).

While the activities of DRN neurons are correlated with a variety of events (Ranade and Mainen, 2009), it appears that reward information is one of the most influential factors that modulate DRN neuronal activity (Bromberg-Martin et al., 2010; Cohen et al., 2015; Inaba et al., 2013; Luo et al., 2015; Miyazaki et al., 2011; Nakamura et al., 2008; Ranade and Mainen, 2009). One of the prevailing questions is whether the DRN is involved in appetitive or aversive information processing. To answer this question, Hayashi et al. (2015) measured the activities of DRN neurons while monkeys performed a Pavlovian conditioning task, in which visual cues predicted appetitive or aversive outcomes. In the ‘appetitive’ trial blocks, conditional visual stimuli which were associated with a liquid reward at different probabilities were presented. In the ‘aversive’ blocks, visual stimuli were associated with an aversive air puff at various probabilities. Note that, this separate presentation of appetitive and aversive conditional stimuli as a block created the appetitive and aversive context. Hayashi et al. (2015) found that single DRN neurons encode both appetitive and aversive information, but over differing time scales: a tonic and categorical activity to discriminate emotional (i.e. appetitive and aversive) contexts, and a relatively phasic, quantitative activity to encode rewarding events (i.e. the probability of outcomes) (Hayashi et al., 2015).

In terms of animal models of psychiatric disorders, recent work using acute pharmacogenetics perturbation on 5-HT neuronal activity of the dorsal and median raphe nuclei has demonstrated that these nuclei are causally linked to differential control on emotional behaviors (Teissier et al., 2015). Specifically, median raphe 5-HT hyperactivity in mice seems to encourage anxiety behavior while low dorsal/median raphe 5-HT activity increases depression-like behavior.

In addition to the raphe nuclei’s heterogeneous populations of 5-HT and non-5-HT neurons and diverse expression of neurotransmitters (and co-transmitters), these neurons also provide diffuse projection to multiple targets, thereby increasing their complexity. The mammalian DRN contains the majority of forebrain-projecting 5-HT neurons (Azmitia and Segal, 1978; Descarries et al., 1975; Jacobs and Azmitia, 1992; Moore et al., 1978; Vertes, 1991), which appears to be highly conserved across vertebrates (Azmitia, 2007). However, the projection of DRN 5-HT neurons is selective. Some 5-HT neurons make collaterals to multiple distal structures, but many appear to selectively target particular areas (Gagnon and Parent, 2014; Vasudeva et al., 2011; Waselus et al., 2011). Glutamatergic inputs appear to be nonuniformly distributed among DR subnuclei (Commons, 2009; Crawford et al., 2011), which could contribute to the diversity of neuronal activity in vivo. 5-HT neurons in different areas are highly interconnected (Bang et al., 2012), which may perhaps be important for autoregulation. Interestingly, GABAergic and glutamatergic inputs to DRN neurons can themselves be regulated by 5-HT (Lemos et al., 2006), which form larger regulatory circuits.

Many GABAergic neurons in the DRN are synaptically connected to 5-HT neurons (Bang and Commons, 2012; Weissbourd et al., 2014). DRN’s GABAergic neurons inhibit at least 20% of midline 5-HT neurons, and are likely important regulators of 5-HT function (Challis et al., 2013). For instance, the corticotropin-releasing factor, a stress-related hormone, can inhibit 5-HT neurons both directly and indirectly (via DRN’s GABAergic neurons) (Kirby et al., 2008; Lowry et al., 2000; Pernar et al., 2004). VGLUT3-expressing (glutamatergic) neurons also form a sizeable population in the DRN (Hioki et al., 2010). Recent studies have suggested a role in reward-based processes (Liu et al., 2014; McDevitt et al., 2014). Interestingly, there appears to be a substantial coexpression of 5-HT and glutamate in many DRN cells, with the potential to regulate targets across multiple timescales (Cohen et al., 2015; Johnson, 1994; Trudeau, 2004). Some of the dopaminergic neurons in DRN can also co-release glutamate and have been shown, with in vivo calcium imaging and optogenetics techniques, to be causally linked to social preference (following isolation) and place avoidance (Matthews et al., 2016).

Animal models with simpler organizations have also been useful to further illuminate the functions of 5-HT on targeted microcircuits (Marder, 2012). For instance, in zebrafish, the habenulo-raphe pathway is found to be necessary for active avoidance learning but not classical fear conditioning (Amo et al., 2014). In *Cancer borealis* crab, two mutually coupled neurons isolated from the gastric mill of the stomatogastric ganglion have been shown to be modulated by 5-HT by increasing the alternating bursting regime in parameter space and burst frequency (Grashow et al., 2009). In the nematode *Caenorhabditis elegans*, 5-HT could for example promote exploitation by speeding up foraging decision-making under complex environments (Iwanir et al., 2016) and transition from crawling to swimming (Vidal-Gadea et al., 2011), while in leeches and lampreys, 5-HT can modulate swimming behavior (Brodfuehrer et al., 1995; Harris-Warrick and Cohen, 1985).

Overall, the picture that has emerged is one of heterogeneity of cell type and connectivity, which has limited strong support for any particular theory of 5-HT function, though there may be some principles of afferent connectivity (Dorocic et al., 2014; Ogawa et al., 2014).

### Neural circuit models

There are several computational models of 5-HT modulation of neural microcircuits. For instance, Meeter et al. (2006) had used a spiking neuronal network model of the hippocampus that included the entorhinal cortex, dentate gyrus, and fields CA1 and CA3, and demonstrated that 5-HT-mediated hyperpolarizing effect on principal cells could affect memory performance. Using simpler ‘firing-rate’-type network models, which included connectivity across the cortex, striatum, DRN, substantia nigra compacta (SNc) and thalamus, Reed et al. (2013) showed that long-range feedback connections in the circuit allows 5-HT to stabilize the network as dopamine neurons get depleted as in Parkinson’s disease. Given the known connections between DRN and the lateral hypothalamus (LHA), Joshi et al. (2011), Jalewa et al. (2014), Joshi (2014), and Joshi et al. (2017) had also developed similar firing-rate type models that described the interactions between the DRN and LHA. In the model in Jalewa et al. (2014), non-5-HT GABAergic neurons in the DRN and LHA were included to study the effects of direct and indirect connectivity on the DRN-LHA circuit dynamics. Using a spiking neuronal network model, which consisted of heterogeneous 5-HT and non-5-HT DRN neurons, Wong-Lin et al. (2011, 2012) accounted for the single-unit neuronal data from non-human primates performing rewarded and unrewarded tasks (Bromberg-Martin et al., 2010), as discussed above. After fitting the data, the model identified a potential DRN microcircuit model architecture that predicted the presence of fast inhibition from the non-5-HT to 5-HT neurons, and slow theta band oscillation in the network.

As the PFC is one of the most densely 5-HT modulated brain regions (Celada et al., 2013), it is important to model and understand the potential effects 5-HT has on the PFC functions. Given that dopamine is also known to strongly innervate the PFC, Wang and Wong-Lin (2013) used (‘mean-field’) firing-rate models to mathematically analyze how dopamine and 5-HT co-modulation on PFC neurons and synapses can affect PFC circuit dynamics. The PFC network model consisted of multiple pyramidal neuronal populations and fast spiking inhibitory interneurons, and NMDA-, AMPA- and GABA-mediated synapses. 5-HT1A, 5-HT2A, dopaminergic D1 and D2 receptor-mediated effects were implemented in the model, constrained by past experimental findings. The network model’s oscillatory behavior was found to be co-modulated in complex, non-intuitive ways, due to the different affinities and the PFC network connectivity. For example, the model showed that certain combination of dopamine and 5-HT receptors could lead to the robustness of beta and gamma band oscillations, or the existence of multiple discrete oscillatory regimes. The model also made predictions in terms of pharmacological (receptor agonist/antagonist) effects. For instance, the model could mimic the effects of 5-HT2A antagonists (by shifting of the input–output function) causing the reduction of beta band oscillation amplitude, which was consistent with 5-HT2 agonist (2,5-dimethoxy-4-iodoamphetamine; DOI) effects in the frontal cortex of anesthetized rats (Budzinska, 2009).

In another work, Cano-Colino et al. (2013, 2014) simulated the effects of 5-HT1A and 5-HT2A receptors on the PFC by incorporating simplified induced currents, I_GIRK_, and modulating membrane currents I_K,Ca_, and I_Can_ and the leak currents, as discussed previously. This was then used to investigate the effects of 5-HT on spatial working memory (SWM). The PFC model, inspired by non-human primate studies (Williams et al., 2002), included inhibitory interneurons and pyramidal neurons, which were used to represent the maintenance of information about different target spatial locations. The model in Cano-Colino et al. (2014) showed that with increasing 5-HT concentration level, SWM performance of the network followed an inverted U-shape manner due to the differential effects of 5-HT1A and 5-HT2A receptors. The model suggested that SWM output errors due to low and high 5-HT levels are caused by the network’s dynamical instabilities. In Cano-Colino et al. (2013), further predictions of the same model were made. Specifically, the model suggested that excessive levels of 5-HT could cause SWM deficits that increased with delay duration, and higher vulnerability to distractors. Interestingly, the neuronal memory fields were predicted to be better tuned than the behavioral report for excessive 5-HT.

Maia and Cano-Colino (2015) also used a similar model to provide an explanation for reduced 5-HT levels leading to obsessive-compulsive disorder (OCD). Specifically, the model suggested that a decrease in 5-HT levels made the network more stable and difficult to get out of a stable steady (‘attractor’) state (arguably a neural substrate for obsessions and compulsions). Simulating the effects of a 5-HT2A blocker (mimicking the partial effects of second-generation antipsychotics) or a 5-HT1A agonist was effective in reducing the OCD state, through decreasing the overall network’s excitability.

Serotonergic influence on synapses has been modeled in a similar way. For example, Puzerey et al. (2014) modeled a cortical (layer 2/3) microcircuit model using excitatory and inhibitory neurons, bombarded by synaptic noise. The network model could mimic the effects of spontaneous epileptic seizures (‘fast runs’) by increasing the synaptic noise (to simulate 5-HT3R-mediated modulation) and excitatory coupling strength (to simulate 5-HT2R-mediated modulation). The inhibition of slow afterhyperpolarization current alone (due to 5-HT2R-mediated modulation) could also induce fast-run oscillation. By increasing the excitatory neuronal leak conductance (to simulate the postsynaptic effect of 5-HT1R activation in the presence of antidepressant fluoxetine) the epileptiform network dynamics can be suppressed, as observed in experiments. To capture a more systemic modulation, the abovementioned Wang and Wong-Lin (2013) work not only modeled the PFC neuronal excitability modulation by 5-HT, but also simultaneously simulated 5-HT modulation on the synapses by varying the excitatory and inhibitory synaptic coupling strengths.

The models discussed here showed how they could be used to systematically and quantitatively study 5-HT modulation on neuronal circuits within and out of the raphe nuclei. Importantly, this type of neural circuit models is promising in terms of mechanistically bridging from neuronal and synaptic physiological and pharmacological level to cognition and behavior.

## Human cognition and behavior

### Human studies

Similar to the animal studies discussed above, 5-HT in humans is known to play a role in a range of processes. They include learning and memory, aggression, sexual behavior, and sleep, among others (Harvey, 2003; Hull et al., 2004; Moresco et al., 2002). Dysfunctions in the 5-HT system have been linked to a range of brain disorders, including depression, anxiety disorders, impulsivity, eating disorders, schizophrenia, and addiction (Dalley et al., 2008, 2011; Maes and Meltzer, 1995; Tanaka et al., 2007; Young, 2013).

5-HT function in humans could be studied with various approaches. They include behavioral neurogenetics (the relationship between genes coding for 5-HT system and behavior), tryptophan depletion (which reduces 5-HT levels synthesized in the brain; see Figure 1), and psychopharmacological (the administration of 5-HT agonists and antagonists to healthy human subjects) studies. These can be combined with brain imaging (e.g. positron emission tomography and functional magnetic resonance imaging; fMRI) (Beliveau et al., 2015; Kumar and Mann, 2014; Spies et al., 2015) and genes related to 5-HT function, (e.g. associated with *SERT* and receptors) (Hariri and Holmes, 2006). In humans, the short allele of this gene has been shown to correlate with harm avoidance behavior compared with individuals with two copies of the long allele (Katsuragi et al., 1999; Lesch et al., 1996; Ricketts et al., 1998). Genetic variation of the 5-HT2A receptor gene is known to influence episodic memory in humans (de Quervain et al., 2003), while the *SERT* gene can affect risk-based decision-making (He et al., 2010; Kuhnen et al., 2013). Many tryptophan depletion studies investigated its role in learning as well as affective processes. For example, modulation in 5-HT levels by acute tryptophan depletion modulates the sensitivity to punishment (for review, Cools et al., 2011). Although controversial, several reports indicate acute tryptophan depletion induces inappropriate switching after probabilistic punishments (i.e. too sensitive to punishments) (Chamberlain, et al., 2006; Cools et al., 2008; Robinson et al., 2012). Psychopharmacological methods can investigate both decrease and increase in 5-HT levels on behavior. For example, 5-HT agonist psilocybin could reduce attentional performance in healthy human patients (Carter et al., 2005). At a more abstract level, 5-HT has also been found to promote prosocial behavior (Siegel and Crockett, 2013).

### Cognitive models

Existing computational cognitive models focus on one or a few of 5-HT’s functions. Many of the models employ high-level reinforcement learning models such as temporal difference learning (Doya, 2002; Huys et al., 2016). There are models that suggest 5-HT plays an antagonistic function with respect to that of dopamine (Asher et al., 2010, 2013; Daw et al., 2002; Weng et al., 2013; Zaldivar et al., 2010). Although there are experimental supports to these studies (Hebart and Glascher, 2015), more recent experimental studies have shown that this theory is incomplete, as there are complex interactions between both neuromodulators (see above). In addition, some 5-HT receptors are found to inhibit dopamine release while others facilitate dopamine release (Alex and Pehek, 2007; Boureau and Dayan, 2011).

Other models have suggested that 5-HT plays more of a role in the scaling of future rewards (Doya, 2002; Schweighofer et al., 2008; Tanaka et al., 2007). Specifically, in these models, 5-HT controls the timing of reward prediction signals, which is represented by the discount factors in reinforcement learning models (Doya, 2002). Functional MRI (Tanaka et al., 2007) and dietary tryptophan depletion (Schweighofer et al., 2008) studies support the role of 5-HT in controlling the timescale of reward prediction as suggested by Doya (2002). A more recent computational modeling work has suggested that 5-HT projections to the striatum plays a role in risk computation, that is, computations related to reward variance (Balasubramani et al., 2014, 2015). These models were able to account for large behavioral datasets, including the role of 5-HT in reward, punishment, and risk-based decision-making, as reported in experimental studies (Homberg, 2012; Long et al., 2009; Murphy et al., 2009; Rogers, 2011).

Overall, cognitive models on 5-HT have been very successful in accounting for various animal and human neural data and decision-making behavior, and their changes due to drugs or psychiatric disorders. These models typically have fewer parameters than neuronal circuit models. However, a challenge would be to bridge it back to the more physiologically constrained neural circuit models.

## Toward bridging across the scales

We have reviewed various experimental and computational approaches, from intracellular to human behavioral levels, and we have seen the complex functional roles played by 5-HT. A major contribution of this review is the synthesis and categorization of a wide range of computational and mathematical models of 5-HT systems. Table 2 provides a summary of the discussed models. Clearly, a number of them are limited to a single level of organization. Hence, an integrated view of 5-HT functions is currently lacking (Nakamura and Wong-Lin, 2014). To make better sense of its multifaceted roles, new approaches are needed.

**Table 2.** Summary of the types of computational models. At each level of organization, models may span across multiple levels of organization. Italics: multiscale models.

Common scale of description	Models	Range of scales	Mathematical/computational descriptions
Single neuronal electrophysiology (membrane potential dynamics)	Tuckwell et al. (2015); Tuckwell and Penington (2014)	Ion channels to neuronal membrane potential	Differential equations
	*Wong-Lin et al. (2011, 2012)*	*Neuronal membrane potential to behavior*	
Presynaptic terminal (5-HT synthesis, release and reuptake)	Best et al. (2010); Stoltenberg and Nag (2010)	Intracellular processes	Differential equations
	*Flower and Wong-Lin et al. (2012)*	*Intracellular processes and neuronal membrane potential*	
	Bunin et al. (1998); Wood et al. (2014)	Release-reuptake	
	*Joshi et al. (2011)*	*Release-reuptake to neural population dynamics*	
Signal transduction & modulated currents	Menziani et al. (2001); Reeves et al. (2003); Schmidt and Peroutka (1989)	Molecule/protein dynamics	Differential equations, and other techniques in physics and chemistry
	Pettigrew et al. (2005); Zhang et al. (2012); Zhou et al. (2014)	Intracellular processes	Differential equations
	Bertram (1993, 1994)	Ion channel currents	
	*Cano-Colino et al. (2014)*	*Ion channel currents to behavior*	
	*Joshi (2014); Joshi et al. (2011; 2017)*	*Release-reuptake to neural population*	
Neural circuit	Jalewa et al. (2014); Joshi et al. (2011); Puzerey et al. (2014); Wang and Wong-Lin (2013)	Microcircuit	Differential equations
	*Cano-Colino et al. (2013, 2014); Maia and Cano-Colino (2015); Meeter et al. (2006); Wong-Lin et al. (2011, 2012)*	*Microcircuit to behavior*	
	*Joshi et al. (2017);* Reed et al. (2013)	Large-scale circuit	
Cognition and behavior	Asher et al. (2013); Balasubramani et al. (2014); Daw et al. (2002); Doya (2002); Weng et al. (2013); Zaldivar et al. (2010)	Behavior	Algorithms, optimal/Bayesian, and difference/differential equations
	*Balasubramani et al. (2015)*	*Neural circuit and behavior*	

5-HT: 5-hydroxytryptamine.

A potential direction would be to develop multiscale computational models that can bridge across multiple levels of descriptions. The models at different scales have to be linked in a consistent way so that the information from a lower scale can be carried into the simplified model at a higher scale. The advantages of such a computational approach are that they can integrate experimental data at various levels and from separate sources, shed insights into the mechanisms across different levels, and form predictions for future experiments.

As an example, suppose there was a research enquiry regarding how the (e.g. oscillatory) dynamics of a neural network is affected in a certain brain function disorder, say major depressive disorder. A biophysically detailed neural network model can reveal how specific intracellular or genetic processes can influence it. Reduced versions of the network model can be rigorously mathematically analyzed and predictions can be made regarding how specific cognition and behavior (e.g. memory) are altered. Multiscale modeling is already used in many fields of natural sciences and engineering (E, 2011; Horstemeyer, 2009) and in biology and systems medicine (Qu et al., 2011; Schnell et al., 2007). However, it has yet to be readily embraced in modeling neuromodulator systems.

Based on our review, we have already seen promising computational models that have bridged across multiple scales (italics in Table 2). For example, we have discussed 5-HT-based computational models that bridge from ion channels through neural networks to cognitive functions (e.g. Cano-Colino et al., 2013, 2014; Wang and Wong-Lin, 2013; Wong-Lin et al., 2011, 2012). Such models can also incorporate neuronal diversity in the DRN (e.g. Wong-Lin et al., 2011, 2012) or targeted brain regions (Cano-Colino et al., 2013, 2014; Wang and Wong-Lin, 2013). Other similar modeling attempts had been focused more on the effects of other monoaminergic systems, namely, dopaminergic and norepinephrine/noradrenaline modulation, especially on cortical computation (e.g. Brunel and Wang, 2001; Durstewitz and Seamans, 2002; Eckhoff et al., 2009, 2011).

One of the key ingredients, and indeed a major challenge when developing multiscale models, is to find essential components or behaviors at each scale that have to be retained and bridge to a higher scale. Otherwise, the overall model will become overwhelming with details, and a conceptual understanding of mechanisms will remain elusive. For instance, when there are multiple temporal scales co-existing, only certain biological variables (e.g. fast variables at the intracellular level of a 5-HT presynaptic terminal) may be important to produce certain behavior (e.g. neuronal activity dependent extracellular 5-HT level) at a higher scale, while leading to a lower dimensional model (e.g. Flower and Wong-Lin, 2014). However, in general it is not always clear cut what components need to be retained or ignored.

An advantage across many of the models discussed is their common mathematical ‘language’, in that they can generally be described by discrete (e.g. state transition algorithms in reinforcement learning) or more often, continuous (e.g. differential equations in neuronal circuit model) dynamical systems (Guckenheimer and Holmes, 1983; Strogatz, 2001). Thus, theories from dynamical systems can provide useful tools to model, analyze and unify various (e.g. emergent) phenomena and concepts in neurobiological and cognitive systems (Finkel, 2000; Freeman, 2007; Rabinovich et al., 2007).

Given this advantage, we may be able to make use of well-established dynamical model reductions methods, (e.g. center manifolds) (Guckenheimer and Holmes, 1983). For example, when certain compositions or dynamical variables of the system operate at relatively slower dynamics, the much faster dynamical variables can be assumed to have reached their quasi-steady states, or averaged out over time and spared the need to be updated over time (e.g. Ermentrout and Terman, 2010; Guckenheimer and Holmes, 1983; Izhikevich, 2007; Roxin and Ledberg, 2008; Wong and Wang, 2006). A simple but effective perturbation technique has been used to elucidate important timescales and strengths of relationships of substrates within the 5-HT presynaptic terminal model, which subsequently led to a reduced low-dimensional model, which can be incorporated into the next (neuronal circuit) scale (Flower and Wong-Lin, 2014). A recent similar technique has also been applied to dopaminergic presynaptic terminals (Cullen and Wong-Lin, 2014, 2015). Thus, well-justified approximations can reduce the number of model parameters and complexity to enhance understanding of the essentials while allowing scalability across multiple scales and with more efficient computations.

Further, ‘mean-field’ techniques, originating from statistical physics (to bridge atomic interactions to thermodynamical properties), have been successfully used for analyzing the emergent dynamical behavior of neural systems (Amit, 1992; Amit et al., 1985; Hopfield, 1982; Wilson and Cowan, 1972, 1973). Mean-field approaches are excellent approximations for understanding stationary states and linear (first-approximation) temporal responses of networks. Extended versions have now been used to investigate behaviors of sufficiently realistic noisy spiking neuronal circuits, providing a means to connect from an ion channel to cognitive models while allowing conceptual understanding via dynamical systems theory (e.g. Brunel and Wang, 2001; Burkitt, 2006a, 2006b; Eckhoff et al., 2011; Hasegawa, 2003; Renart et al., 2003). For example, Wang and Wong-Lin (2013) showed that it is possible to perform rigorous mathematical (stability) analysis of a system of heterogeneous populations of cortical neurons co-modulated differently by 5-HT and dopamine. In another example, Joshi et al. (2017) proposed a promising mean-field modeling framework to bridge to large-scale brain circuit dynamics, which can link pharmacological mechanisms to neuroimaging studies. However, more advanced mean-field approaches that embrace heterogeneity in the system (e.g. Harrison et al., 2015; Ly, 2015; Nichola and Campbell, 2013) would be required to model the complex and heterogeneous 5-HT system.

Crucial to the development of computational models and theoretical approaches are the availability of accurate experimental data. Despite decades of research linking 5-HT with its various functions such as emotional control, we still do not know what information 5-HT neurons signal nor how such signals are encoded. There are two reasons for such slow progress: (i) there is a lack of methods to record the activity of identified 5-HT neurons during behavior, and previous attempts are largely based on neuronal electrophysiological characteristics; and (ii) recent work has shown that the midbrain raphe is more complex than anticipated, with multiple 5-HT neuron subtypes. Interactions within the raphe microcircuitry also remain poorly understood.

To overcome the above issues, technical advances to tag neurons with chemical and anatomical specificity would lead to further understandings of the roles of specific types of neurons in the raphe (Cohen et al., 2015; Liu et al., 2014). Advanced data analytical (e.g. optogenetic) tools to manipulate serotonergic neurons would help refine current theories of their functions (Cohen et al., 2015; Fonseca et al., 2015; Liu et al., 2014; McDevitt et al., 2014; Miyazaki et al., 2014). Capturing the 5-HT system’s complexity will require large-scale recordings of identified neurons in behaving animals, and the generation of large datasets. Advanced analytical data (e.g. machine learning) techniques, modeling, and interpretation of such datasets will be required (Gao and Ganguli, 2015; Helmstaedter, 2015; Huys et al., 2016). In human studies, elucidating the connectivity changes due to 5-HT perturbations (e.g. drugs or gene alleles) could illuminate the whole-brain changes on behavior. Hence, effective/functional connectivity analysis of global brain activity such as dynamic causal modeling, Granger causal analysis and machine learning may prove useful (e.g. Beliveau et al, 2015; Youssofzadeh et al., 2015a, 2015b).

In summary, we have reviewed current experimental and computational work on 5-HT across multiple scales. We have also suggested that a more holistic understanding of the 5-HT’s complex functions may require an integrated multiscale modeling approach. With the availability of such multiscale models, one can rapidly test hypotheses, and provide model predictions that can be verified by future experiments. Such an approach could also illuminate future treatments related to brain disorders involving serotonergic dysfunctions, including anxiety, depression, schizophrenia and post-traumatic stress disorder. All of these will depend not only on advanced theoretical and computational techniques, but also on advanced experimental methods to generate better datasets and the systematic curation of the data.
